# Supplementary material for: A first-principles phase field method for quantitatively predicting multi-composition phase separation without thermodynamic empirical parameter
Source: Nat Commun. 2019 Aug 1;10:3451. doi: 10.1038/s41467-019-11248-z (PMC6671953; doi:10.1038/s41467-019-11248-z)
Supplement: Supplementary file 1 — Supplementary Information [file 41467_2019_11248_MOESM1_ESM.pdf]

Supplementary Information

A first-principles phase field method for quantitatively predicting multi-composition phase separation without thermodynamic empirical parameter

Bhattacharyya et al.

Supplementary Figures:

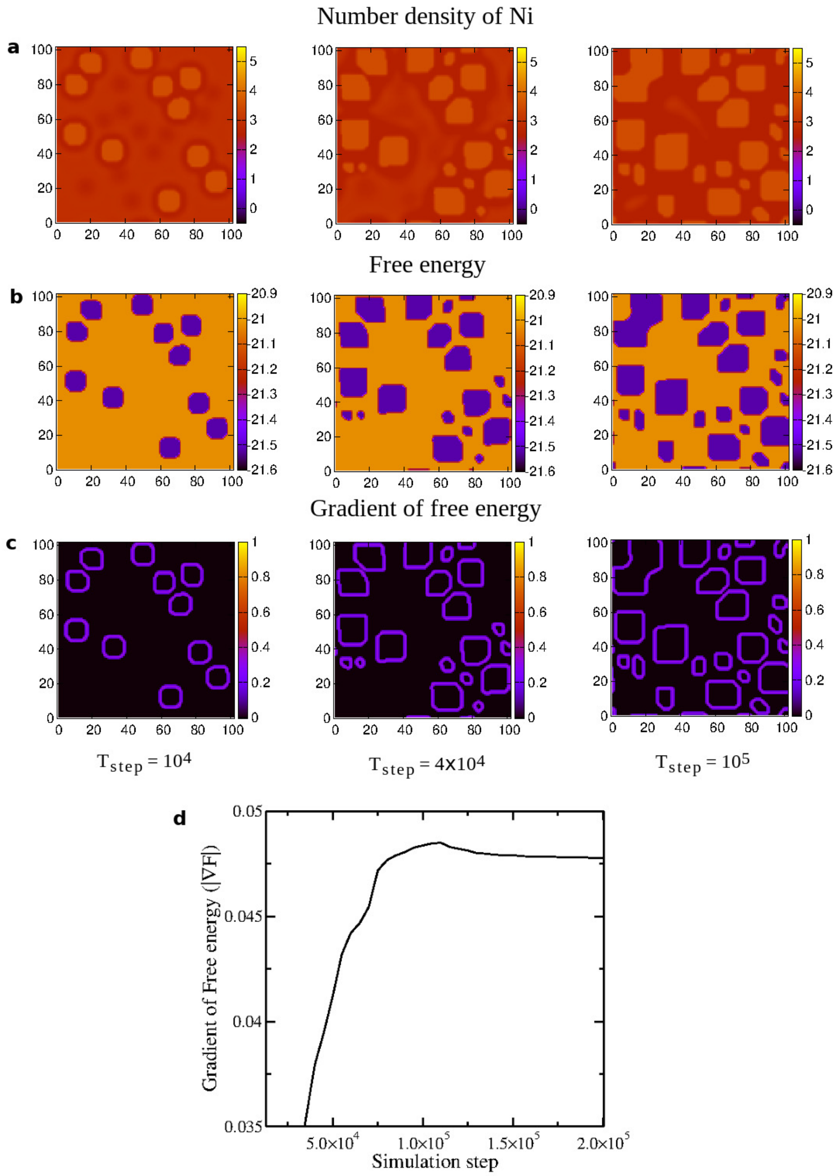

Supplementary Fig. 1: **Change in free energy gradient with simulation time.** 2D plot of (**a**) number density of Ni, (**b**) free energy and (**c**) magnitude of free energy gradient for Ni 60% at various simulation time. (**d**) The plot of magnitude of free energy gradient, summed over all space as a function of simulation time step

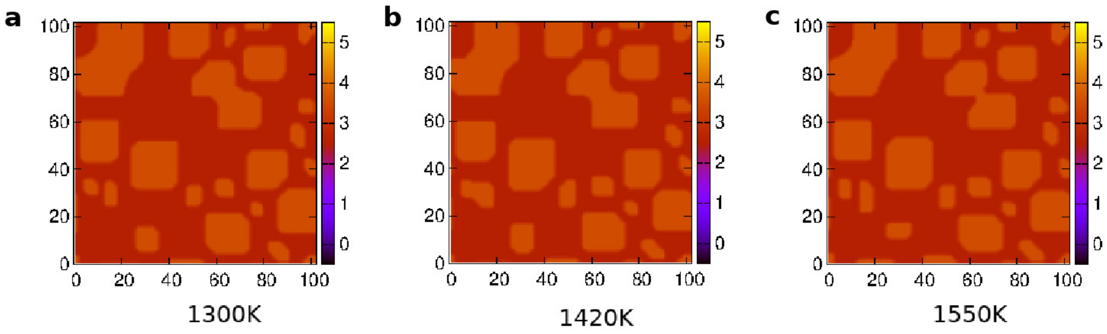

Supplementary Fig. 2: **Effect of temperature on microstructure.** Plot of φ_{Ni} at time t=1.6×10^5 for composition $Ni_{2.4}Al_{1.6}$ (Ni 60%) at temperatures (**a**)1300K (**b**)1420K and (**c**) 1550K. There is reduction in the bright phase with increase in temperature.

Supplementary Note 1: Estimation of gradient energy coefficient (ε_{X})

We compare the second term of Eq.(3) in our paper with the previously reported PFM to get an estimation of ε_X. This term corresponds to the contribution from the interface energy to the chemical potential. In the conventional PFM, the total free energy of microstructure is written in terms of the field variable (φ) as,

$$F = \int_V \left[f(\varphi) + \frac{\varepsilon}{2}(\nabla\varphi)^2 \right] dV, \tag{1}$$

where, f is the local free energy density and ε denotes the gradient energy coefficient of the field variable[1]. Taking partial derivative with respect to φ on both sides of Eq.(1) we get,

$$\frac{\delta}{\delta\varphi} F = \frac{\delta}{\delta\varphi} \int_V f(\varphi) dV - \varepsilon\nabla^2\varphi. \tag{2}$$

This equation is now similar to that of the definition of chemical potential (Eq.(3) in our paper). If we convert the value of ε_X from a dimensionless one to the proper unit, using the estimation of Δx from the length scale of the experimental microstructure, then it will become equivalent to the gradient energy coefficient of the PFM.

We use Eq.(6) and Eq.(17) to calculate 2D and 3D Laplacian, respectively in the last terms of Eqs.(2a), (2b), and Eq.(3) in our paper numerically. We set $\varepsilon_X = 0.5$ for our simulation and the estimated value of Δx from the experimental microstructure of Ref.[2] is 0.03μm, giving $\frac{\varepsilon_X}{(\Delta x)^2} = 5.56 \times 10^2$ eV/μm. Then the expression within the parenthesis of the right hand side of the above equation will give the numerical representation of $\nabla^2\varphi_X$ (with unit of μm^{-2}) for $\Delta x = 1\mu$m. Now, the real value of ε_X is calculated to be 5.56 $\times 10^2$ eV/μm. Converting the units to the SI system, the

gradient energy coefficient of our calculation is approximately 8.9×10^{-11}J/m, which matches very closely to the gradient energy coefficient (6.0×10^{-11}J/m) used in Ref.[1] for conventional PFM.

Supplementary Note 2: Potential renormalization calculation

We have used Vienna Ab initio Simulation Package (VASP)[3,4] for *ab-initio* density functional theory calculation to obtain energies of the tetrahedron clusters in the cluster expansion theory. We have performed spin polarized calculation for the clusters having Ni atoms. The projector augmented-wave method [5,6] is used. The electron exchange and correlation energy is treated within the Perdew-Burke-Ernzerhof (PBE)[7] generalized gradient approximation (GGA). The plane wave kinetic energy cut-off is determined as 400 eV. A $8 \times 8 \times 8$ k-point sampling are introduced. Lattice constants are set as the average of the optimized pure Ni and Al, 3.78 Å.

For the first-step potential renormalization calculations, we define three dimensional grid points in the lattice around the central atom. Using lattice symmetry we identify the irreducible grid points. We construct atomic positions by changing the central atom coordinates to each of these grid points and keeping all other atoms at fixed positions. Thus we get one set of atomic positions corresponding to each grid point. These sets of atomic positions are used in the input files (POSCAR) for VASP. We get total energies for each of these input atomic position set.

Next we estimate the renormalization corrections per one tetrahedron with 4 atoms. A fortran code, `Boltzweight-pureM.f` is written for this purpose. Each input file, corresponding to an alloy composition, contains the serial number (arbitrary), total energy per supercell (32 atom system), number of degeneracy (C_i). A sample input file `data-A4B0.dat` is enclosed in the accompanied code. This file contains data for Ni_4. In the program, the parameter `radius` corresponds to the radius of the cube to numerically integrate the partition function. Here, the value

e.g., `radius=0.09d0` corresponds to 0.09 times the supercell size.

`ZZ` and `FF` are the partition function and the local free energy, respectively. The renormalization effect of displacements of the center atom is shared by a number of surrounding tetrahedrons. Therefore, the value, `FF`, is finally divided by this number to obtain the renormalization correction per one tetrahedron with 4 atoms. In Ref.[8], Misumi et. al., divided ΔF (`FF`) by 8. However, we have observed that deviding it by 8 is inappropriate. A more suitable procedure is to divide ΔF by the number of effective surrounding tetrahedrons, M, that do not share edge or point. To find M we calculate the number of surrounding atoms including the centre atom and divide it by 4. There are 12 nearest neighbor (n.n.) atoms and the center atom multiplied by M should be added to it. Then we solve the equation $(12 + M)/4 = M$, to get $M = 4$. To find M in bcc structures, the total number of atoms in the effective surrounding tetrahedrons is calculated as $8 + M$, where 8 represents n.n. atoms to the center atom. In bcc, the surrounding tetrahedrons are not regular tetrahedrons but are distorted ones with two different edge lengths (one is the n.n. distance and the other is the second n.n distance). These distorted tetrahedrons have 3 n.n. atoms including the center atom. Therefore, we devide the number of atoms by 3 and solve the equation $(8 + M)/3 = M$ to get $M = 4$. Fortran codes for potential renormalization calculation of bcc structure are included in the folder `BW-bcc`.

In the program, we can obtain the local free energy values of the input data for the temperatures ranging from 10K to 2300K for an increment of 10 K. Depending on the number of grids and radius, the finally obtained renormalization corrections may be slightly changed numerically.

Supplementary Note 3: Explanation to use the accompanied phase field model code

The code calls for three input files as explained one by one below.

1. *input*: This file contains the main input parameters for our phase field model calculation.

Below is an example of the input file, with explanation for each entry:

```
2           :: Enter 2 for 2D and 3 for 3D simulation
1.7         :: Initial concentration of Ni (φ_Ni in the code)
3.3         :: Initial concentration of Al (φ_Al in the code)
6.0         :: Radius of the seeds
0.8d0       :: The grid space, Δx
0.00125d0   :: The time step, Δt (M_x = 1)
0.5         :: ε_X, the gradient energy coefficient
0           :: Time step from where to restart the simulation
1000        :: Output files are written after an interval of this value
10000       :: Maximum time step
128         :: Number of grid point in x direction
128         :: Number of grid point in y direction
1           :: Number of grid point in z direction (1 for 2D simulation)
0.3         :: Amplitude of the seeds, the c parameter
0.5         :: Amplitude for random fluctuation
0.05        :: Coefficient for the polynomial in free energy boundary
```

2. *in_para*: This file lists the free energy values of all the clusters from cluster expansion theory and the potential renormalisation theory. Values for Ni-Al alloy at 1300K are listed in Table 1 of the paper. The first number is total composition, i.e., $n + m$ in Ni_nAl_m cluster. Next an array of numbers are written for dimensions, (i, j) for $i, j = 0, 1, 2...(n + m)$. The $(i, j)^{th}$ term in the array (i.e., the ith row and j^{th} column) corresponds to the energy of Ni_iAl_j cluster. We have used up to $(n + m) = 6$ for our calculations. This input file is constant for a type of alloy at a specific temperature. Here is our *in_para* file for Ni-Al alloy (fcc lattice) at 1300K.

```
6
0.0        -1.895      -6.030    -10.475  -14.113  -9.132   14.509
-1.717     -7.515      -12.896   -17.523  -16.539  3.540    19.217
-7.099     -14.1274    -20.433   -23.262  -8.151   8.257    24.689
-13.852    -21.447     -24.924   -11.402  -3.431   13.766   130.0
-21.250    -25.393     -14.684   -7.627   1.996    130.0    170.0
-26.168    -14.458     -9.204    -1.928   130.0    170.0    220.0
-14.964    -10.0166    -3.990    90.0     150.0    200.0    240.0
```

(We put some arbitrary large values for the points where $(i + j) \geq (m + n)$. These values have no effect on the final microstructures for $(m + n) \geq 5$)

3. *coordinate*: This file contains the coordinates of the initial seeds in the uniform matrix for the input microstructure. The first line gives the number of seeds. The fractional coordinates are written the next line onwards. The list of coordinates has different format for 2D and 3D calculation. For 2D calculation, three columns as x coordinate, y coordinate and sign of the seed

has to be written. The positive sign assigns Ni-rich composition to the seed and the negative sign assigns Al-rich seeds. For the 3D simulation, z coordinates of the seeds must be specified in the third column as shown in the following examples.

Example of `coordinate` file for 2D simulation:

```
4
0.69803922   0.65098039    1.00
0.90333333   0.23544954    1.00
0.43137255   0.12549020   −1.00
0.55686275   0.40000000   −1.00
```

Example of `coordinate` file for 3D simulation:

```
2
0.69803922   0.65098039   0.500    1.00
0.48627451   0.33333333   0.500   −1.00
```

Finite difference method for Laplacian operator

2 dimensional (function NABLA2): $\varphi(i, j)$ can be expressed using the nearest and next nearest neighbour grid points in a 2D mesh [9]. Taylor series expansion of $\varphi(i, j)$ gives,

$$\varphi(i \pm 1, j) = \varphi(i, j) \pm \frac{\partial \varphi(i, j)}{\partial x}\Delta x + \frac{1}{2}\frac{\partial^2 \varphi(i, j)}{\partial x^2}\Delta x^2, \tag{3}$$

$$\varphi(i, j \pm 1) = \varphi(i, j) \pm \frac{\partial \varphi(i, j)}{\partial y}\Delta y + \frac{1}{2}\frac{\partial^2 \varphi(i, j)}{\partial y^2}\Delta y^2, \tag{4}$$

$$\varphi(i \pm 1, j \pm 1) = \varphi(i, j) \pm \frac{\partial \varphi(i, j)}{\partial x}\Delta x + \frac{1}{2}\frac{\partial^2 \varphi(i, j)}{\partial x^2}\Delta x^2 \pm \frac{\partial \varphi(i, j)}{\partial y}\Delta y + \frac{1}{2}\frac{\partial^2 \varphi(i, j)}{\partial y^2}\Delta y^2. \tag{5}$$

Assuming $\Delta x = \Delta y$, we added $\varphi(i+1, j)$ and $\varphi(i-1, j)$ from Eq.3 to the sum of $\varphi(i, j+1)$ and $\varphi(i, j-1)$ from Eq.4. Adding $\varphi(i+1, j+1)$, $\varphi(i+1, j-1)$, $\varphi(i-1, j+1)$ and $\varphi(i-1, j-1)$, from Eq.(5) weighted by $1/2$ to the previous summation we get,

$$\nabla^2\varphi(i,j) = \frac{1}{\Delta x^2}\Big(\frac{1}{2}\Big[\varphi(i+1,j)+\varphi(i-1,j)+\varphi(i,j+1)+\varphi(i,j-1)\Big] + \frac{1}{4}\Big[\varphi(i+1,j+1)+\varphi(i+1,j-1)+\varphi(i-1,j+1)+\varphi(i-1,j-1)\Big] - 3\varphi(i,j)\Big) + O(\Delta x)^2. \tag{6}$$

3 dimensional (function NABLA3) We have extended the above formalism to three dimension. The Tailor expansion of $\varphi(i, j, k)$ will give the following sets of equations,

$$\varphi(i\pm 1,j,k) = \varphi(i,j,k) \pm \frac{\partial\varphi(i,j,k)}{\partial x}\Delta x + \frac{1}{2}\frac{\partial^2\varphi(i,j,k)}{\partial x^2}\Delta x^2, \tag{7}$$

$$\varphi(i,j\pm 1,k) = \varphi(i,j,k) \pm \frac{\partial\varphi(i,j,k)}{\partial y}\Delta y + \frac{1}{2}\frac{\partial^2\varphi(i,j,k)}{\partial y^2}\Delta y^2, \tag{8}$$

$$\varphi(i,j,k\pm 1) = \varphi(i,j,k) \pm \frac{\partial\varphi(i,j,k)}{\partial z}\Delta z + \frac{1}{2}\frac{\partial^2\varphi(i,j,k)}{\partial z^2}\Delta z^2, \tag{9}$$

$$\varphi(i\pm 1,j\pm 1,k) = \varphi(i,j,k) \pm \frac{\partial\varphi(i,j,k)}{\partial x}\Delta x + \frac{1}{2}\frac{\partial^2\varphi(i,j,k)}{\partial x^2}\Delta x^2 \pm \frac{\partial\varphi(i,j,k)}{\partial y}\Delta y + \frac{1}{2}\frac{\partial^2\varphi(i,j,k)}{\partial y^2}\Delta y^2, \tag{10}$$

$$\varphi(i,j\pm 1,k\pm 1) = \varphi(i,j,k) \pm \frac{\partial\varphi(i,j,k)}{\partial y}\Delta y + \frac{1}{2}\frac{\partial^2\varphi(i,j,k)}{\partial y^2}\Delta y^2 \pm \frac{\partial\varphi(i,j,k)}{\partial z}\Delta z + \frac{1}{2}\frac{\partial^2\varphi(i,j,k)}{\partial z^2}\Delta z^2, \tag{11}$$

$$\varphi(i\pm 1,j,k\pm 1) = \varphi(i,j,k) \pm \frac{\partial\varphi(i,j,k)}{\partial x}\Delta x + \frac{1}{2}\frac{\partial^2\varphi(i,j,k)}{\partial x^2}\Delta x^2 \pm \frac{\partial\varphi(i,j,k)}{\partial z}\Delta z + \frac{1}{2}\frac{\partial^2\varphi(i,j,k)}{\partial z^2}\Delta z^2, \tag{12}$$

$$\varphi(i\pm 1,j\pm 1,k\pm 1) = \varphi(i,j,k) \pm \frac{\partial\varphi(i,j,k)}{\partial x}\Delta x + \frac{1}{2}\frac{\partial^2\varphi(i,j,k)}{\partial x^2}\Delta x^2 \pm \frac{\partial\varphi(i,j,k)}{\partial y}\Delta y + \frac{1}{2}\frac{\partial^2\varphi(i,j,k)}{\partial y^2}\Delta y^2 \pm \frac{\partial\varphi(i,j,k)}{\partial z}\Delta z + \frac{1}{2}\frac{\partial^2\varphi(i,j,k)}{\partial z^2}\Delta z^2. \tag{13}$$

Let us assume that $\Delta x = \Delta y = \Delta z$. Adding the positive and negative versions of Eq.(7), 8 and 9 and rearranging, we get,

$$\nabla^2 \varphi(i,j,k) = \frac{1}{\Delta x^2}\Big[\varphi(i+1,j,k) + \varphi(i-1,j,k) + \varphi(i,j+1,k) + \varphi(i,j-1,k) + \varphi(i,j,k+1) + \varphi(i,j,k-1) - 6\varphi(i,j,k)\Big]. \tag{14}$$

Similarly adding positive and negative versions of Eqs.(10), (11) and (12) we obtain Eq.(15) and from Eq.(13) we get Eq.(16) as follows.

$$\begin{aligned}\nabla^2 \varphi(i,j,k) = \frac{1}{4\Delta x^2}\Big[&\varphi(i+1,j+1,k) + \varphi(i+1,j-1,k) + \varphi(i-1,j+1,k) + \varphi(i-1,j-1,k)\\ &+ \varphi(i,j+1,k+1) + \varphi(i,j+1,k-1) + \varphi(i,j-1,k+1) + \varphi(i,j-1,k-1)\\ &+ \varphi(i+1,j,k+1) + \varphi(i+1,j,k-1) + \varphi(i-1,j,k+1) + \varphi(i-1,j,k-1)\\ &- 12\varphi(i,j,k)\Big]\end{aligned} \tag{15}$$

and

$$\begin{aligned}\nabla^2 \varphi(i,j,k) = \frac{1}{4\Delta x^2}\Big[&\varphi(i+1,j+1,k+1) + \varphi(i+1,j+1,k-1) + \varphi(i+1,j-1,k+1)\\ &+ \varphi(i+1,j-1,k-1) + \varphi(i-1,j+1,k+1) + \varphi(i-1,j+1,k-1)\\ &+ \varphi(i-1,j-1,k+1) + \varphi(i-1,j-1,k-1) - 8\varphi(i,j,k)\Big].\end{aligned} \tag{16}$$

Adding Eqs.(14), (15) and (16) and then rearranging we get the final form to calculate Lapla-

cian numerically.

$$\begin{aligned}\nabla^2\varphi(i,j,k) = \Big[&\varphi(i+1,j,k)+\varphi(i-1,j,k)+\varphi(i,j+1,k)+\varphi(i,j-1,k)+\varphi(i,j,k+1)+\varphi(i,j,k-1)\\ &+\frac{1}{4}\Big(\varphi(i+1,j+1,k)+\varphi(i+1,j-1,k)+\varphi(i-1,j+1,k)+\varphi(i-1,j-1,k)\\ &+\varphi(i,j+1,k+1)+\varphi(i,j+1,k-1)+\varphi(i,j-1,k+1)+\varphi(i,j-1,k-1)\\ &+\varphi(i+1,j,k+1)+\varphi(i+1,j,k-1)+\varphi(i-1,j,k+1)+\varphi(i-1,j,k-1)\\ &+\varphi(i+1,j+1,k+1)+\varphi(i+1,j+1,k-1)+\varphi(i+1,j-1,k+1)\\ &+\varphi(i+1,j-1,k-1)+\varphi(i-1,j+1,k+1)+\varphi(i-1,j+1,k-1)\\ &+\varphi(i-1,j-1,k+1)+\varphi(i-1,j-1,k-1)\Big)-11\varphi(i,j,k)\Big]\frac{1}{3\Delta x^2}\\ &+O(\Delta x)^2.\end{aligned} \tag{17}$$

Time stepping

We use the forward differentiating method for time step calculations as follows:

$$\frac{\partial\varphi(i,j,k)}{\partial t} = \frac{\varphi^{n+1}(i,j,k)-\varphi^{n}(i,j,k)}{\Delta t}. \tag{18}$$

Pseudocode For the subroutine `phasefield`

```
if(flag=2) perform 2D simulation:
1. Calculate and write free energy in the file Free.dat.
2. Initialization of idum (random seed) for random number generation in gasdev()
3. Construct initial microstructure for φNi(i,j) and φAl(i,j) as follows,
   The uniform matrix:
   for i,j:=1,Nx do
      φNi= X_ini                   !X_ini, Y_ini  are read from input
      φAl= Y_ini
     The n seeds:
      for ii:=1,n do
        Form circles of radius R (read from input) centered at each
        coordinate, having the following values for φNi(i,j) and φAl(i,j)
        φNi=X_ini+sign*c        !sign is in the last column of coordinate file
        φAl=Y_ini-sign*c
      end for
   Calculate free energy,
    PHI_free(i,j)= F_en(φNi,φAl)    !F_en(x,y) gives free energy as per Fig.4(b)
   end for
 4. If restart file is available read the initial values of φNi and φAl
     Calculate free energy,
```

PHI_free(i,j)= F_en(φ_{Ni},φ_{Al})

5. Add all the values in φ_{Ni} and divide it by (Nx*Ny) to get the average φ_{Ni} Repeat the process for φ_{Al} and free energy.

6. Write the initial values of φ_{Ni}, φ_{Al} and free energy in `out_Ni_0`, `out_Al_0` and `out_free_0`

7. Start time step:

```
for t_step:=1,tmax do
      apply periodic boundary to φNi and φAl
      apply laplacian to φNi and φAl
  for i,j:=1,Nx do
    Calculate chemical potential:
      dF_enX = df_enX + F_en(φNi+0.5,φAl) − F_en(φNi−0.5,φAl) + random force
      dF_enY = df_enY + F_en(φNi,φAl+0.5) − F_en(φNi,φAl−0.5) + random force
    Add the interface energy contribution
      μNi=dF_enX−εx∇²φNi
      μAl=dF_enY−εx∇²φAl
  end for
  apply periodic boundary to μNi and μAl
  apply laplacian to μNi and μAl
  proceed one time step:
  for i,j:=1,Nx do
     φNi(t + Δt) = φNi(t) + ΔtM∇²μNi
     φAl(t + Δt) = φAl(t) + ΔtM∇²μAl
```

```
        calculate free energy:
          PHI_free(i,j)=F_en(φNi,φAl)
    end for
   check for divergence in φNi or φAl -> terminate if any
   write following output files at an interval (specified in input)
   out_Ni_(timestep), out_Al_(timestep) and out_free_(timestep)
  end for
8. Calculate average values for φNi, φAl and free energy and
   write into output file avg_phi
else if(flag=3) do 3D simulation:
   Same as above No. 1 to 8.
   The arrays have dimensions (Nx,Ny,Nz).
   Only φNi is written in out_Ni_3D_(timestep) for faster simulation.
else if(flag=any other value)
   Show error massage
end if
```

Supplementary References

1. Zhu, J. *et al.* Three-dimensional phase-field simulations of coarsening kinetics of γ' particles in binary Ni-Al alloys. *Acta Materialia* **52**, 2837–2845 (2004).

2. Lee, H.-Y., Demura, M., Xu, Y., Wee, D.-M. & Hirano, T. Selective dissolution of the γ phase in a binary Ni(γ)/$Ni_3Al(\gamma')$ two-phase alloy. *Corros. Sci.* **52**, 3820–3825 (2010).

3. Kresse, G. & Hafner, J. *Ab initio* molecular dynamics for liquid metals. *Phys. Rev. B* **47**, 558–561 (1993).

4. Kresse, G. & Furthmüller, J. Efficient iterative schemes for ab initio total-energy calculations using a plane-wave basis set. *Phys. Rev. B* **54**, 11169–11186 (1996).

5. Blöchl, P. E. Projector augmented-wave method. *Phys. Rev. B* **50**, 17953–17979 (1994).

6. Kresse, G. & Joubert, D. From ultrasoft pseudopotentials to the projector augmented-wave method. *Phys. Rev. B* **59**, 1758–1775 (1999).

7. Perdew, J. P., Burke, K. & Ernzerhof, M. Generalized gradient approximation made simple. *Phys. Rev. Lett.* **77**, 3865–3868 (1996).

8. Misumi, Y., Masatsuji, S., Sahara, R., Ishii, S. & Ohno, K. A lattice Monte Carlo simulation of the FePt alloy using a first-principles renormalized four-body interaction. *J. Chem. Phys.* **128**, 234702 (2008).

9. Provatas, N. & Elder, K. Appendix B: Basic Numerical Algorithms for Phase Field Equations. In *Phase-Field Methods in Materials Science and Engineering*, 262–263 (Wiley-Blackwell, 2010), 1st edn.
