# Supplementary figures and images for: A first-principles phase field method for quantitatively predicting multi-composition phase separation without thermodynamic empirical parameter
Source: Nat Commun. 2019 Aug 1;10:3451. doi: 10.1038/s41467-019-11248-z (PMC6671953; doi:10.1038/s41467-019-11248-z)

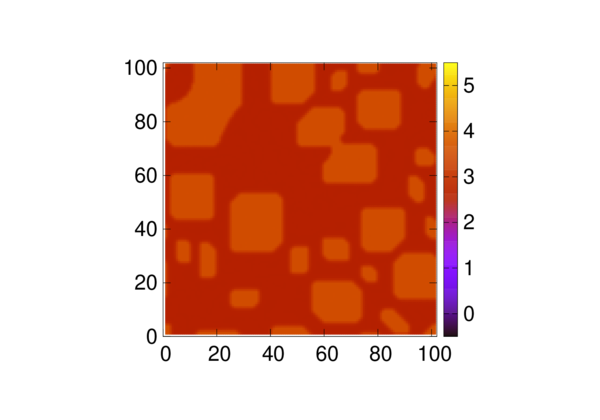

Supplement: Supplementary file 3 — Source Data [file 41467_2019_11248_MOESM3_ESM.zip › supplementary-files/data-files/Fig.S2/FigS2-c-1550K/out_Ni_160000.eps.png]
